# Characterization of the complete mitochondrial genome and phylogenetic analysis of *Tetraselmis marina* (Cienkowski) R.E.Norris, Hori & Chihara 1980

**DOI:** 10.1080/23802359.2026.2657109

**Published:** 2026-04-20

**Authors:** Changhong Cheng, Cheng-Hao Chen, Fangfang Yang

**Affiliations:** a Nansha Islands Coral Reef Ecosystem National Observation and Research Station/Key Laboratory of South China Sea Fishery Resources Exploitation & Utilization, Ministry of Agriculture and Rural Affairs, South China Sea Fisheries Research Institute, Chinese Academy of Fishery Sciences, China; b South China Sea Ecological Center, MNR, China; c Key Laboratory of Tropical Marine Bio-resources and Ecology, South China Sea Institute of Oceanology, Chinese Academy of Sciences, Guangzhou, China

**Keywords:** *Tetraselmis marina*, mitogenome, phylogeny

## Abstract

*Tetraselmis marina* (Cienkowski) R.E.Norris, Hori & Chihara 1980, a marine green microalga, holds considerable value in aquaculture due to its well-rounded nutritional profile. Gaining insights into the mitochondrial genome of this species is crucial for advancing studies on its phylogeny and evolutionary relationships. In the present research, we for the first time characterized the complete mitochondrial genome of *T. marina*. This genome forms a circular structure with a total length of 51,196 base pairs (bp), encompassing 34 protein-coding genes, 24 transfer RNA (tRNA) genes, and 3 ribosomal RNA (rRNA) genes. Nucleotide composition analysis showed a relatively balanced distribution: adenine (A) accounted for 32.9%, thymine (T) for 33.8%, guanine (G) for 16.5%, and cytosine (C) for 16.8%. Phylogenetic investigations demonstrated that *T. marina* is classified within the Chlorodendraceae family and shares the closest evolutionary affinity with *Tetraselmis sp*. CCMP 881.

## 1. Introduction

*Tetraselmis marina* (Cienkowski) R.E.Norris, Hori & Chihara 1980, a coastal green microalga within the genus *Tetraselmis* (family Chlorodendraceae), acts as a vital nutritional source for aquatic organisms, primarily due to its elevated fatty acid content (Moussa et al. 2017). Multiple species of *Tetraselmis* have attracted considerable interest on account of their remarkable nutrient removal efficiency and halotolerance (Herold et al. 2021; Magpusao et al. 2021). Previous research has underscored *T. marina*’s capacity to boost lipid or carotenoid biosynthesis under nitrogen or phosphorus repletion regimes (Moussa et al. 2017), along with its effectiveness in eliminating water contaminants (Petroutsos et al. 2008; Cameron et al. 2018). Distinguishing between different *Tetraselmis* strains poses a challenge, and mitochondrial genomes play a pivotal role in elucidating evolutionary genomics, phylogenetics, and population genetics of these organisms. Despite its ecological and biotechnological importance, the mitochondrial genome of *T. marina* had not been investigated prior to this work. In the current study, we sequenced and annotated the complete mitochondrial genome of *T. marina*, and these findings are anticipated to furnish valuable insights into the genetic characteristics and evolutionary processes of this species.

## 2. Materials and methods

**Figure 1.**
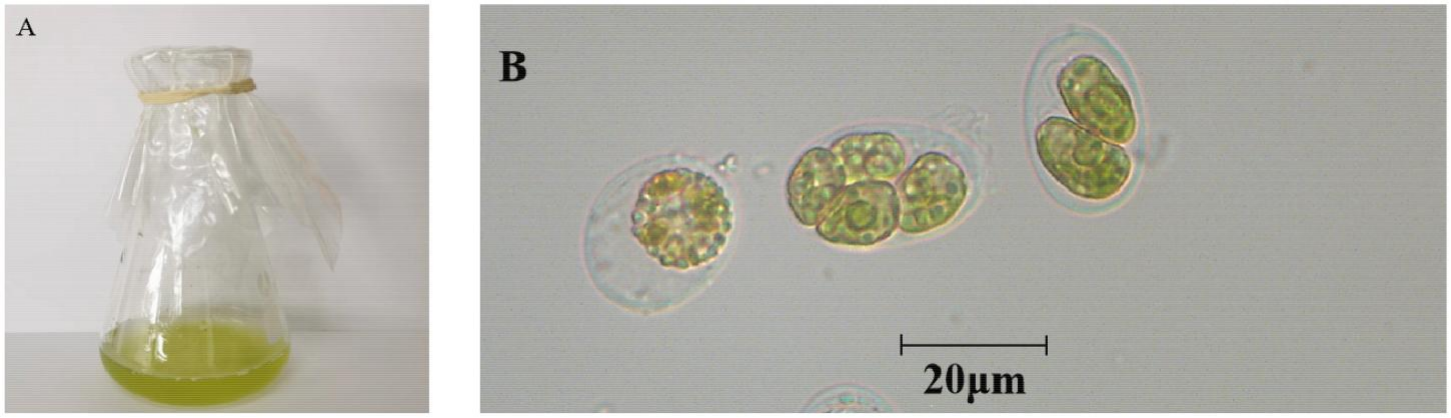
The morphology of *T. marina*: (A) in flask cultures and (B) under microscopic observation, with photographs captured by Fangfang Yang.

*T. marina* used in this study was originally isolated from the coral *Pocillopora damicornis* collected from the marine area of Sanya, Hainan Province, China (18°18′ N, 109°48′ E), and gently rinsed with sterile seawater. Subsequently, a WaterpikTM device was employed to collect the tissue slurry containing *T. marina*. Species identification of *T. marina* was performed based on morphological characteristics and phylogenetic analysis of conserved mitochondrial genes. The microalga was then separated from the tissue slurry *via* single-cell isolation method and subsequently cultivated in f/2 medium. The cultures were maintained in the laboratory of the South China Sea Fisheries Research Institute, Guangzhou City, Guangdong Province (www.scsfri.ac.cn/; Chenghong Cheng, chengchanghong09@163.com) with a voucher number of CC311 (Figure 1).

Genomic DNA extraction was carried out using the Plant Genomic DNA Kit (Tiangen Biotech, Beijing, China) in strict accordance with the manufacturer’s recommended protocol. Thereafter, the complete mitochondrial genome was obtained *via* whole-genome sequencing using the Illumina Novaseq 6000 Sequencing Platform (Illumina, CA, USA), with an average sequencing coverage depth exceeding 200× (Figure S1). Raw sequencing data were subjected to quality control (QC) filtering using fastp v0.36 software. De novo assembly of the circular mitochondrial genome was performed from high-quality paired-end reads using the SPAdes 3.14.1 program with a K-mer size of 105. Annotation of the mitochondrial genome was accomplished using MFannot and Mitofy software with default parameters (Allio et al. 2020), adopting the standard genetic code. The physical map of the complete mitochondrial genome was generated using PMGview (http://www.1kmpg.cn/pmgview) (Liu et al. 2023). For phylogenetic analysis, mitochondrial genome sequences of 12 additional species were retrieved from GenBank to construct a phylogenetic tree. Multiple sequence alignment of 25 conserved protein-coding genes across the complete mitochondrial genomes of all 13 species (including *T. marina*) was performed using MAFFT 7.037 with the FFT-NS-2 algorithm (Katoh and Standley 2016). The 25 conserved protein-coding genes (PCGs) used for multiple sequence alignment are as follows: *atp1*, *atp4*, *cox1*, *cox2*, *cox3*, *nad1*, *nad2*, *nad3*, *nad4*, *nad4L*, *nad5*, *nad6*, *nad10*, *rpl5*, *rpl6*, *rpl10*, *rpl16*, *rps4*, *rps12*, *rps19*, *tatC*, *rps8*, *rps11*, *rpl2*, and *rpl14*. The optimal evolutionary model (cpREV + F+R3) was determined using ModelFinder v1.6 (Kalyaanamoorthy et al. 2017). Maps of genes with cis- and trans-spliced introns were generated using PMGmap tool (Zhang et al. 2024). Finally, the Maximum Likelihood (ML) phylogenetic tree was constructed with 1,000 bootstrap replicates using IQ-TREE 2.0 software (Minh et al. 2020).

## 3. Results

**Figure 2.**
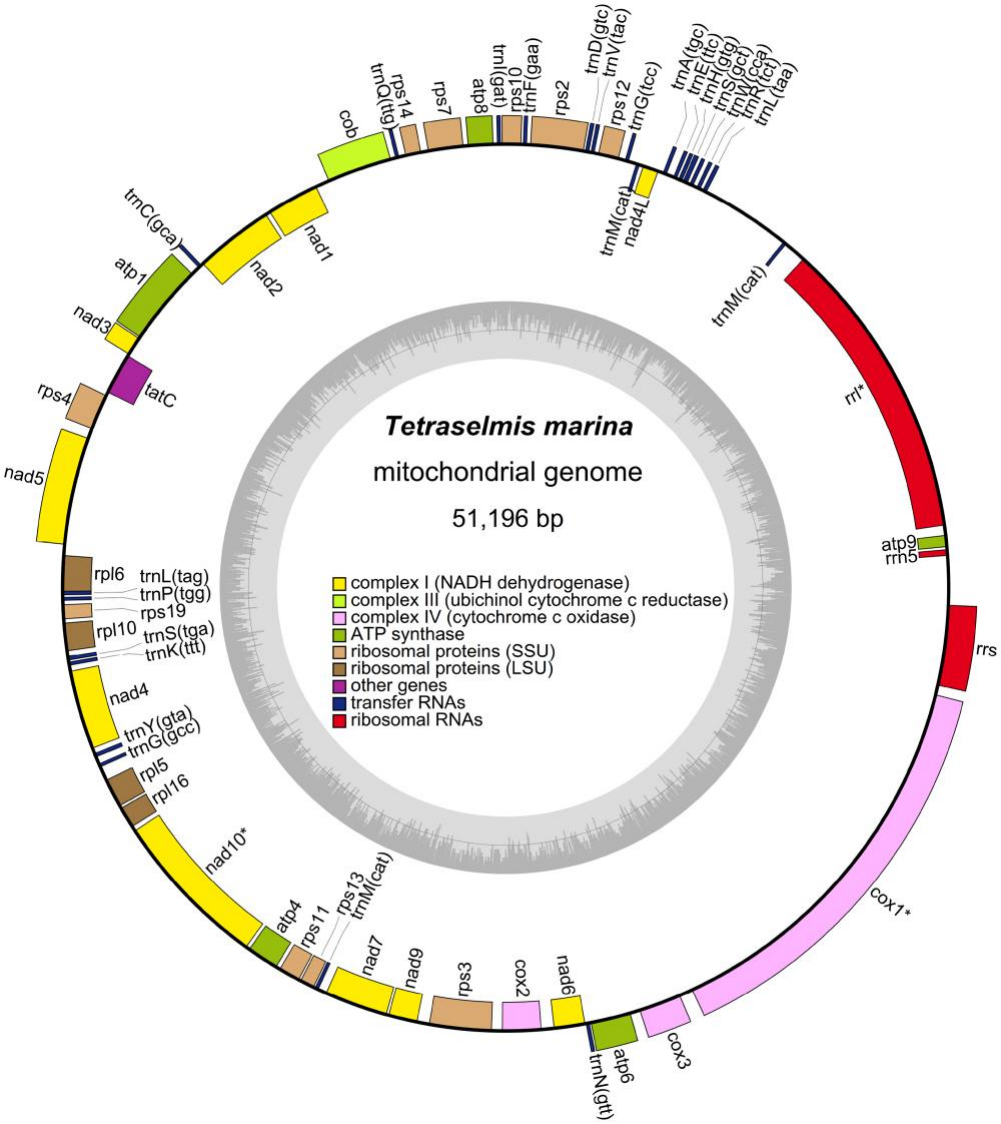
The complete mitochondrial genome map of *T. marina* illustrates the organization of 61 genes, comprising 34 PCGs, 24 tRNA genes, and 3 rRNA genes. Circular structure explanation: the outer circle represents the positive strand of the mitochondrial genome, and the inner circle represents the negative strand. Color block definitions: Red = complex I (NADH dehydrogenase, e.g. *nad1*, *nad2*); orange = complex III (ubiquinol cytochrome c reductase); blue = complex IV (cytochrome c oxidase, e.g. *cox1*, *cox2*); green = ATP synthase (e.g. *atp1*, *atp4*); purple = ribosomal proteins (small subunit, SSU; e.g. *rps4*, *rps12*); dark blue = ribosomal proteins (large subunit, LSU; e.g. *rpl5*, *rpl6*); yellow = other genes (e.g. *tatC*); light green = transfer RNAs (tRNAs, labeled with their corresponding amino acid and anticodon, e.g. *trnM*(cat)); gray = ribosomal RNAs (rRNAs: rrs = small subunit rRNA, *rrl* = large subunit rRNA). Genes marked with an asterisk (*) indicate cis-splicing genes (*nad10*, *cox1*).

**Figure 3.**
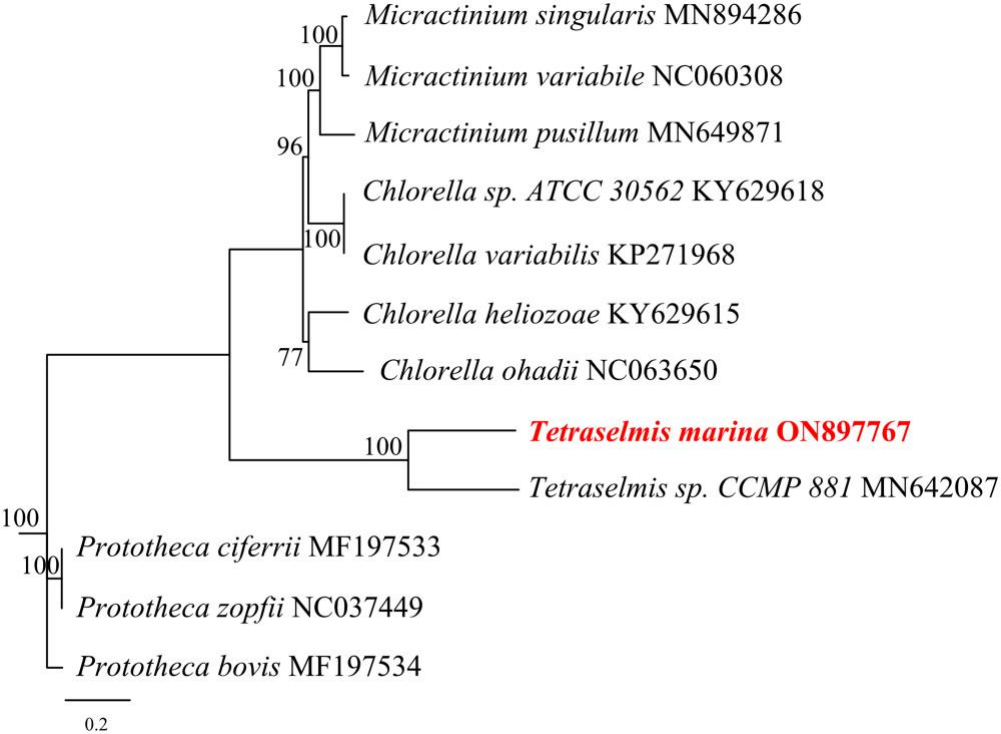
Phylogenetic tree generated using the maximum-likelihood method based on the complete mitochondrial genomes of *T. marina* and 11 additional closely related species. The sequences incorporated in this analysis are as follows: *T. marina* (ON897767), *Tetraselmis* sp. CCMP 881 (MN642087.1) (Turmel et al. 2020), *Chlorella variabilis* (KP271968.1) (Orsini et al. 2016), *Chlorella* sp. ATCC 30562 (KY629618.1) (unpublished), *Chlorella heliozoae* (KY629615.1) (unpublished), *Chlorella ohadii* (NC063650) (unpublished), *Micractinium variabile* (NC060308) (unpublished), *Micractinium singularis* (MN894286) (unpublished), *Micractinium pusillum* (MN649871) (Kang et al. 2019), *Prototheca zopfii* (NC037449) (unpublished), *Prototheca bovis* (MF197534) (unpublished), and *Prototheca ciferrii* (MF197533) (unpublished).

The complete mitochondrial genome of *T. marina* has been deposited in GenBank with the accession number ON897767. This circular genome spans 51,196 base pairs, harboring 34 protein-coding genes (PCGs), 24 transfer RNA (tRNA) genes, and 3 ribosomal RNA (rRNA) genes (Figure 2). The overall nucleotide composition was as follows: 32.9% adenine (A), 33.8% thymine (T), 16.5% guanine (G), and 16.8% cytosine (C). Notably, among the 34 PCGs, *rps12* initiated with the start codon GTG, *nad2* with TTG, while all other PCGs utilized ATG as the start codon. In terms of stop codons, *tatC*, *nad5*, *rpl5*, and *cox3* terminated with TAG, whereas the remaining PCGs ended with TAA. Additionally, the genes *rrl*, *nad10*, and *cox1* were identified as cis-splicing genes (Figure S2), and no trans-spliced genes were detected in the mitochondrial genome of *T. marina*. Phylogenetic analysis revealed that *T. marina* and *Tetraselmis sp*. CCMP 881 formed a monophyletic clade (Figure 3), indicating a close evolutionary relationship between the two species.

## 4. Discussion and conclusion

This study reported the first comprehensive characterization of the mitochondrial genome of *T. marina*, providing critical insights into the genetic architecture and evolutionary relationships of this ecologically and biotechnologically important marine microalga. The circular mitogenome of *T. marina* (51,196 bp) exhibits distinct features conserved within the genus *Tetraselmis*, including a high AT content (66.7%) and a gene composition (34 PCGs, 24 tRNAs, 3 rRNAs) congruent with closely related taxa (Turmel et al. 2020). Such conservation underscores the utility of mitogenomic traits for resolving phylogenetic relationships within *Tetraselmis* and supports the taxonomic placement of *T. marina* in the Chlorodendraceae family. The identification of cis-splicing genes (rrl, nad10, and cox1) and the absence of trans-spliced genes in *T. marina*’s mitogenome add to our understanding of gene structure evolution in green microalgae, as cis-splicing is a relatively conserved feature among chlorophytes and may reflect functional constraints on mitochondrial gene expression.

Phylogenetic analysis based on 25 conserved PCGs strongly supports the monophyly of *T. marina* and *Tetraselmis sp*. CCMP 881, confirming their close evolutionary affinity. This high node support indicates that the selected conserved PCGs are robust markers for resolving intra-generic relationships within *Tetraselmis*. Among other taxa included in the analysis, the clade comprising *Micractinium* sp. and *Chlorella* sp. also exhibits strong statistical support, aligning with existing taxonomic frameworks that group these genera within Trebouxiophyceae. Furthermore, the longer mitogenome length of *T. marina* (51,196 bp) compared to other *Tetraselmis* species raises questions about the evolutionary drivers of genome expansion in this genus. Understanding the functional implications of genome size variation would also enhance our appreciation of *T. marina*’s ecological role.

In summary, this study provides a valuable genetic resource for T*. marina* and advances our understanding of *Tetraselmis* phylogenetics. The high support for intra-generic clades validates the use of mitogenomic data for resolving relationships within this genus, while the conserved AT content and gene structure highlight evolutionary constraints in *Tetraselmis* mitogenomes. This study will also be critical for refining the classification of Chlorodendraceae and improving our understanding of green microalga diversification.

## Supplementary Material

s.doc

## Data Availability

The genome sequence data that support the findings of this study are openly available in GenBank of NCBI at (https://www.ncbi.nlm.nih.gov/) under the accession no. ON897767. The associated BioProject, SRA, and Bio-Sample numbers are PRJNA844566, SRR19521163, and SAMN28834016, respectively.
