# Supplementary material for: Characterization of the complete mitochondrial genome and phylogenetic analysis of Tetraselmis marina (Cienkowski) R.E.Norris, Hori & Chihara 1980
Source: Mitochondrial DNA B Resour. 2026 Apr 20;11(5):636–9. doi: 10.1080/23802359.2026.2657109 (PMC13097178; doi:10.1080/23802359.2026.2657109)
Supplement: s.doc [file TMDN_A_2657109_SM5293.doc]

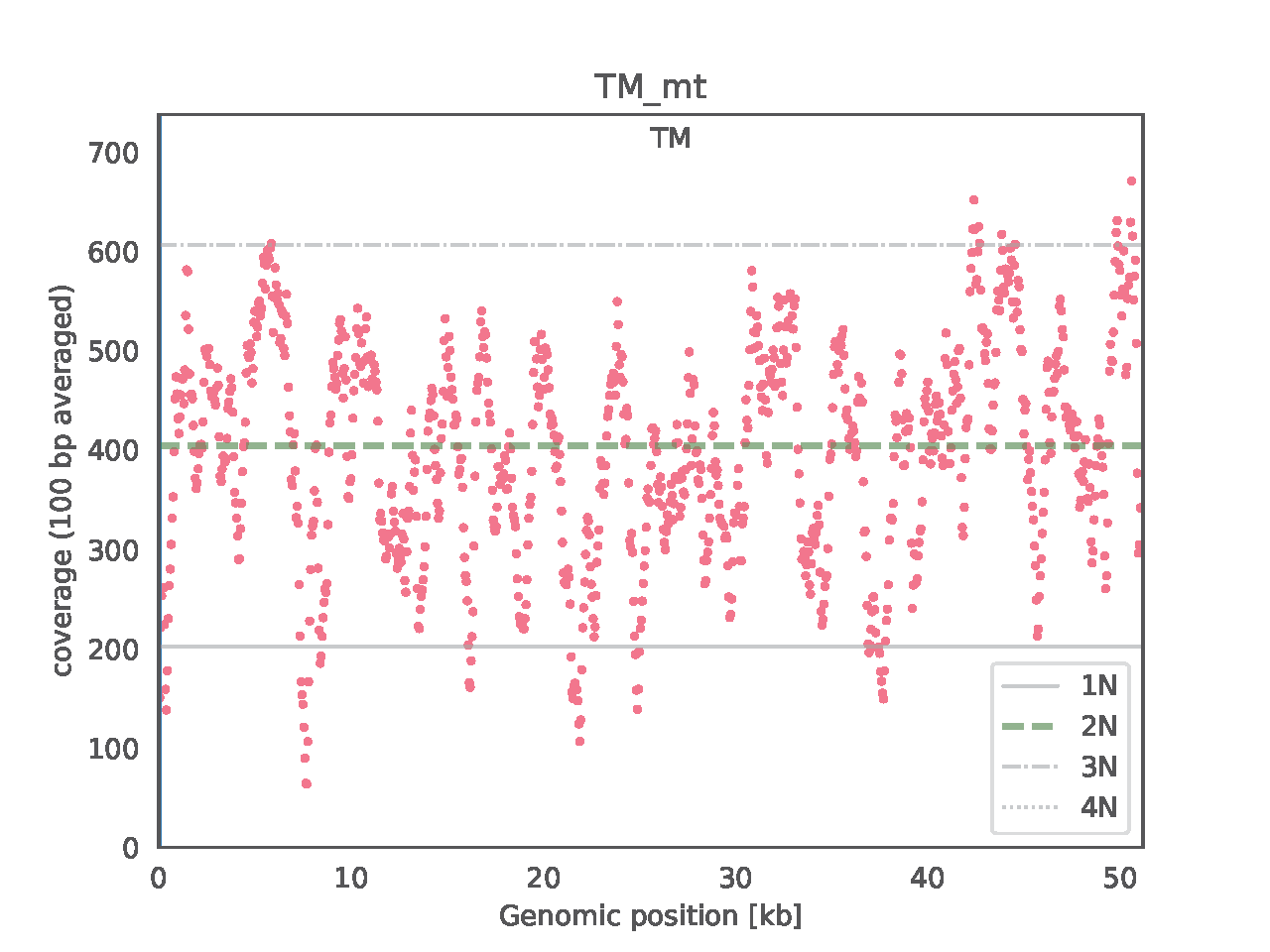


Figure S1 Sequencing coverage depth of the *T. marina* mitochondrial genome. Symbols and metrics: The x-axis represents the position of the mitochondrial genome (bp); the y-axis represents the coverage depth (number of reads mapped to each position). Data points (red dots): Each dot corresponds to the average coverage depth of a single 100-bp window across the genome.

*
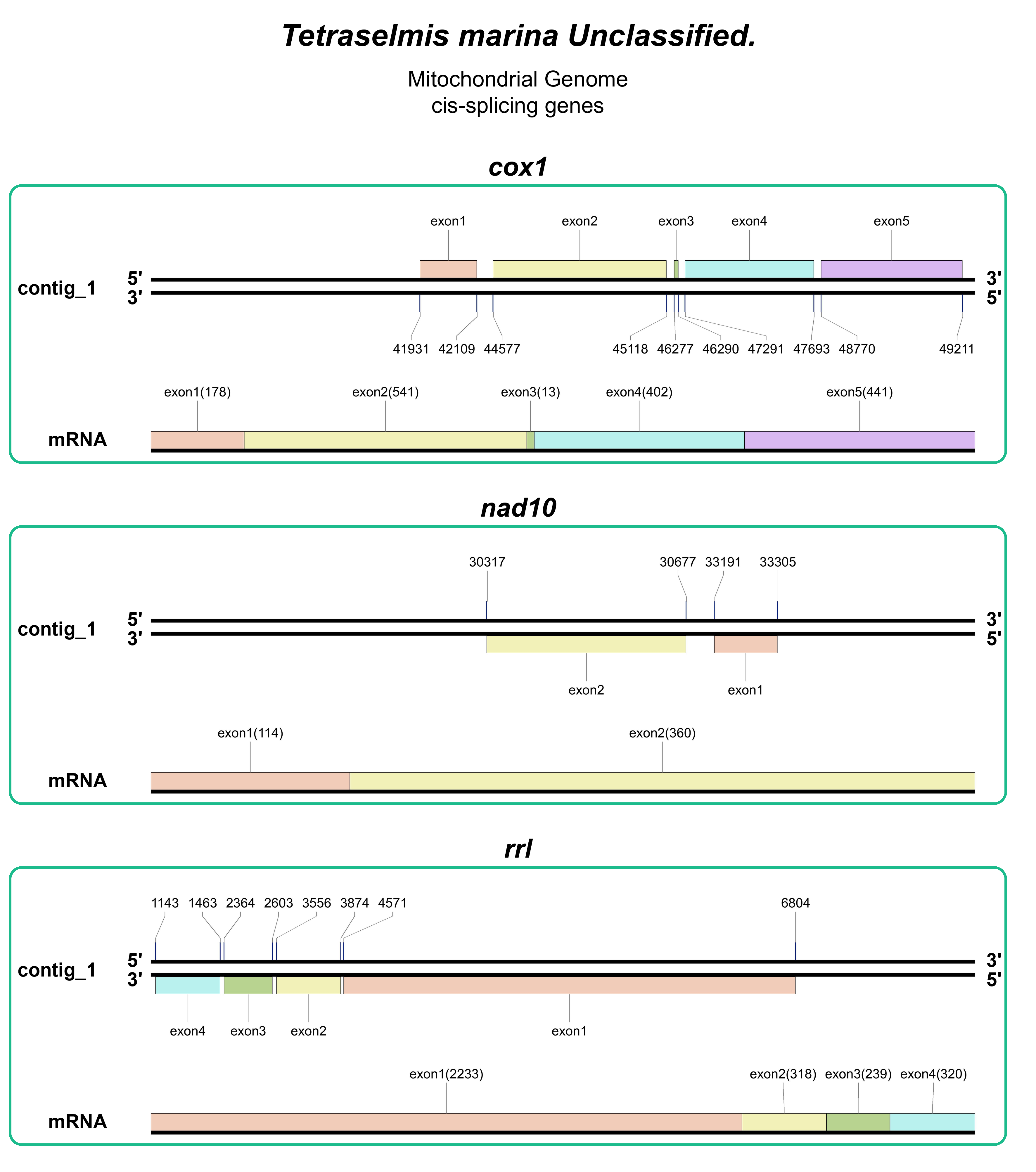
*

Figure S2 Schematic map of the cis-splicing genes in the Tetraselmis marina complete mitochondrial genome. ON897767 definition.
